# Supplementary material for: Phase-Selective Microwave Assisted Synthesis of Iron(III) Aminoterephthalate MOFs
Source: Materials (Basel). 2020 Mar 23;13(6):1469. doi: 10.3390/ma13061469 (PMC7142456; doi:10.3390/ma13061469)
Supplement: Supplementary file 1 [file materials-13-01469-s001.pdf]

materials

MDPI

Article

Phase-Selective Microwave Assisted Synthesis of Iron(III) Aminoterephthalate MOFs

Ana Arenas-Vivo [1], David Avila [2], Patricia Horcajada [1,*]

[1] Advanced Porous Materials Unit, IMDEA Energy. Av. Ramón de la Sagra 3, 28935 Móstoles-Madrid, Spain; ana.arenas@imdea.org

[2] Department of Inorganic Chemistry, Chemical Sciences Faculty, Complutense University of Madrid, 28040 Madrid, Spain; davilabr@ucm.es

* Correspondence: patricia.horcajada@imdea.org

1. Microwave-assisted solvothermal synthesis

Table S1. Letter codes for the resulting products of the following MW synthesis.

Letter Code	Resulting Reaction Product
A	MIL-53(Fe)-NH_2
B	MIL-88B(Fe)-NH_2
C	MIL-101(Fe)-NH_2
X	Amorphous
D	Fe_2O_3

Table S2. Mass, mol and molar ratios, and dispensed amounts for the MW investigation of the system $FeCl_3{\cdot}6H_2O/H_2BDC\text{-}NH_2/HCl$ in water (V H_2O = 4 mL, T = 100 °C, t = 5 min). The resulting phases are indicated by the letter assigned in Table S1.

Sample Name	$FeCl_3{\cdot}6H_2O$ (mg)	$FeCl_3{\cdot}6H_2O$ (mmol)	H_2BDC-NH_2 (mg)	H_2BDC-NH_2 (mmol)	Ligand: Metal	[Fe] (M)	HCl 1 M (mL)	HCl: Fe	Result*
MW 1-01	21.6	0.08	14.48	0.08	1	0.02	0	0	C
MW 1-02	54	0.2	36.2	0.2	1	0.05	0	0	C + A
MW 1-03	108	0.4	72.4	0.4	1	0.1	0	0	A + C
MW 1-04	216	0.8	144.8	0.8	1	0.2	0	0	A
MW 1-05	54	0.2	36.2	0.2	1	0.05	0.05	0.25	C + A
MW 1-06	54	0.2	36.2	0.2	1	0.05	0.1	0.5	C + A
MW 1-07	54	0.2	36.2	0.2	1	0.05	0.15	0.75	A + C
MW 1-08	54	0.2	36.2	0.2	1	0.05	0.2	1	A

* In the case of mixture, the first letter is the major phase

Table S3. Mass, mol and molar ratios, and dispensed amounts for the MW investigation of the system $FeCl_3{\cdot}6H_2O/H_2BDC\text{-}NH_2/HCl$ in water (V H_2O = 4 mL, T= 150 °C, t= 5 min and (•) t= 30 min). The resulting phases are indicated by the letter assigned in Table S1.

Sample Name	$FeCl_3$ $6H_2O$ (mg)	$FeCl_3$ $6H_2O$ (mmol)	H_2BDC-NH_2 (mg)	H_2BDC-NH_2 (mmol)	Ligand: Metal	[Fe] (M)	HCl 1 M (mL)	HCl: Fe	Result *
MW 2-01	21.6	0.08	14.48	0.08	1	0.02	0	0	C + A
MW 2-02	54	0.2	36.2	0.2	1	0.05	0	0	A + C
MW 2-03	108	0.4	72.4	0.4	1	0.1	0	0	A
MW 2-04(•)	108	0.4	72.4	0.4	1	0.1	0	0	A
MW 2-05	216	0.8	144.8	0.8	1	0.2	0	0	A
MW 2-06	54	0.2	36.2	0.2	1	0.05	0.1	0.5	C + A

* In the case of mixture, the first letter is the major phase

Table S4. Mass, mol and molar ratios, and dispensed amounts for the MW investigation of the system $FeCl_3{\cdot}6H_2O/H_2BDC\text{-}NH_2$ in ethanol (V EtOH = 4 mL. T= 150 °C. t= 5 min). The resulting phases are indicated by the letter assigned in Table S1.

Sample name	$FeCl_3{\cdot}6H_2O$ (mg)	$FeCl_3$ $6H_2O$ (mmol)	H_2BDC-NH_2 (mg)	H_2BDC-NH_2 (mmol)	Ligand: Metal	[Fe] (M)	HCl 1 M (mL)	HCl: Fe	Result
MW 3-01	21.6	0.08	14.48	0.08	1	0.02	0	0	B
MW 3-02	54	0.2	36.2	0.2	1	0.05	0	0	B
MW 3-03	108	0.4	72.4	0.4	1	0.1	0	0	B
MW 3-04	216	0.8	144.8	0.8	1	0.2	0	0	B

Table S5. Mass, mol and molar ratios, and dispensed amounts for the MW investigation of assigned in Table S1. the system $FeCl_3{\cdot}6H_2O/H_2BDC\text{-}NH_2$ in ethanol (V EtOH = 4 mL. T= 150 °C. t= 5 min). The resulting phases are indicated by the letter assigned in Table S1.

Sample Name	$FeCl_3{\cdot}6H_2O$ (mg)	$FeCl_3$ $6H_2O$ (mmol)	H_2BDC-NH_2 (mg)	H_2BDC-NH_2 (mmol)	Ligand: Metal	[Fe] (M)	HCl 1 M (mL)	HCl: Fe	Result
MW 3-05	216	0.8	36.2	0.2	0.25	0.2	0	0	B
MW 3-06	216	0.8	72.4	0.4	0.5	0.2	0	0	B
MW 3-07	216	0.8	217.2	1.2	1.5	0.2	0	0	B
MW 3-08	216	0.8	289.6	1.6	2	0.2	0	0	B

Table S6. Mass, mol and molar ratios, and dispensed amounts for the MW investigation of the system $FeCl_3{\cdot}6H_2O$/H_2BDC-NH_2 in ethanol (V EtOH = 4 mL, [Fe] =0.1 M, ligand:metal = 1:1). The resulting phases are indicated by the letter assigned in Table S1.

Sample Name	$FeCl_3{\cdot}6H_2O$ (mg)	$FeCl_3$ $6H_2O$ (mmol)	H_2BDC-NH_2 (mg)	H_2BDC-NH_2 (mmol)	Ligand: Metal	[Fe] (M)	T (°C)	Time (min)	Result
MW 3-09	108	0.4	72.4	0.4	1	0.1	100	5	B
MW 3-10	108	0.4	72.4	0.4	1	0.1	100	10	B
MW 3-11	108	0.4	72.4	0.4	1	0.1	100	20	C
MW 3-12	108	0.4	72.4	0.4	1	0.1	100	30	C
MW 3-13	108	0.4	72.4	0.4	1	0.1	150	10	B
MW 3-14	108	0.4	72.4	0.4	1	0.1	150	20	B
MW 3-15	108	0.4	72.4	0.4	1	0.1	150	30	B
MW 3-16	108	0.4	72.4	0.4	1	0.1	180	5	B
MW 3-17	108	0.4	72.4	0.4	1	0.1	180	10	D
MW 3-18	108	0.4	72.4	0.4	1	0.1	180	20	D
MW 3-19	108	0.4	72.4	0.4	1	0.1	180	30	D

Table S7. Mass, mol and molar ratios, and dispensed amounts for the MW investigation of the system $FeCl_3{\cdot}6H_2O$/H_2BDC-NH_2 in DMF (V DMF = 4 mL. T = 150 °C. ligand:metal = 1:1). The resulting phases are indicated by the letter assigned in Table S1.

Sample Name	$FeCl_3$ $6H_2O$ (mg)	$FeCl_3$ $6H_2O$ (mmol)	H_2BDC-NH_2 (mg)	H_2BDC-NH_2 (mmol)	Ligand: Metal	[Fe] (M)	T (°C)	Time (min)	Result*
MW 4-01	21.6	0.08	14.48	0.08	1	0.02	150	5	X
MW 4-02	54	0.2	36.2	0.2	1	0.05	150	5	C + B
MW 4-03	108	0.4	72.4	0.4	1	0.1	150	5	B + C
MW 4-04	216	0.8	144.8	0.8	1	0.2	150	5	C + A
MW 4-05	21.6	0.08	14.48	0.08	1	0.02	150	30	C
MW 4-06	54	0.2	36.2	0.2	1	0.05	150	30	C + B
MW 4-07	108	0.4	72.4	0.4	1	0.1	150	30	B + C
MW 4-08	216	0.8	144.8	0.8	1	0.2	150	30	A + C

* In the case of mixture, the first letter is the major phase

Table S8. Mass, mol and molar ratios, and dispensed amounts for the MW investigation of the system $FeCl_3{\cdot}6H_2O/H_2BDC\text{-}NH_2/HCl$ in DMF (V DMF = 4 mL. T = 150 °C. t = 5 min). The resulting phases are indicated by the letter assigned in Table S1.

Sample Name	$FeCl_3$ $6H_2O$ (mg)	$FeCl_3$ $6H_2O$ (mmol)	H_2BDC-NH_2 (mg)	H_2BDC-NH_2 (mmol)	Ligand: Metal	[Fe] (M)	HCl 1 M (mL)	HCl: Fe	Result*
MW 4-10	54	0.2	36.2	0.2	1	0.05	0	0	C + B
MW 4-11	54	0.2	36.2	0.2	1	0.05	0.1	0.5	B
MW 4-12	54	0.2	54.3	0.3	1.5	0.05	0	0	C + B
MW 4-13	54	0.2	54.3	0.3	1.5	0.05	0.1	0.5	B

* In the case of mixture, the first letter is the major phase

2. PXRD patterns

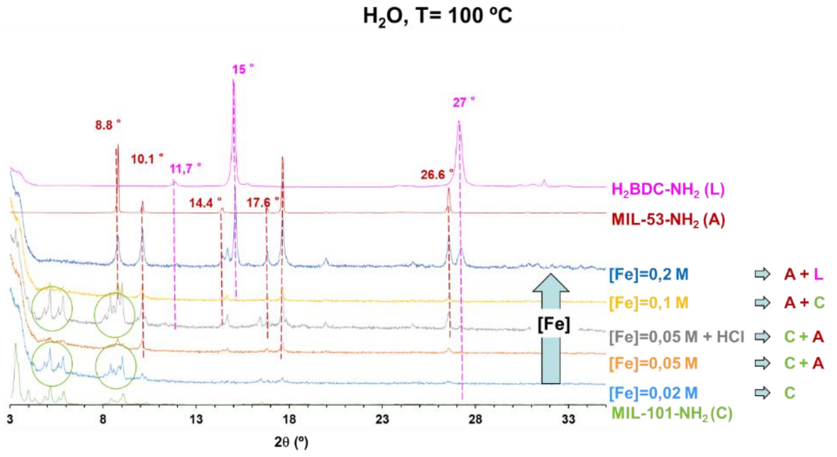

Figure S1. PXRD patterns for the MW investigation of the system $FeCl_3{\cdot}6H_2O/H_2BDC\text{-}NH_2/HCl$ in water (V H_2O = 4 mL, T = 100 °C, t = 5 min) after activation with EtOH, compared to simulated MIL-101-NH_2 (green), MIL-53-NH_2 (red) and H_2BDC-NH_2 (pink).

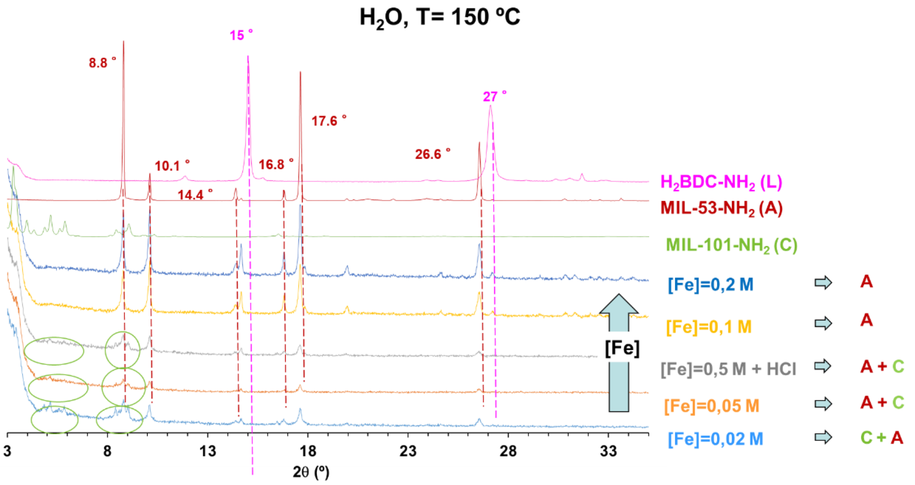

Figure S2. PXRD patterns for the MW investigation of the system $FeCl_3{\cdot}6H_2O/H_2BDC\text{-}NH_2/HCl$ in water (V H_2O = 4 mL, T = 150 °C, t = 5 min) after activation with EtOH, compared to simulated MIL-101-NH_2 (green), MIL-53-NH_2 (red) and H2BDC-NH_2 (pink).

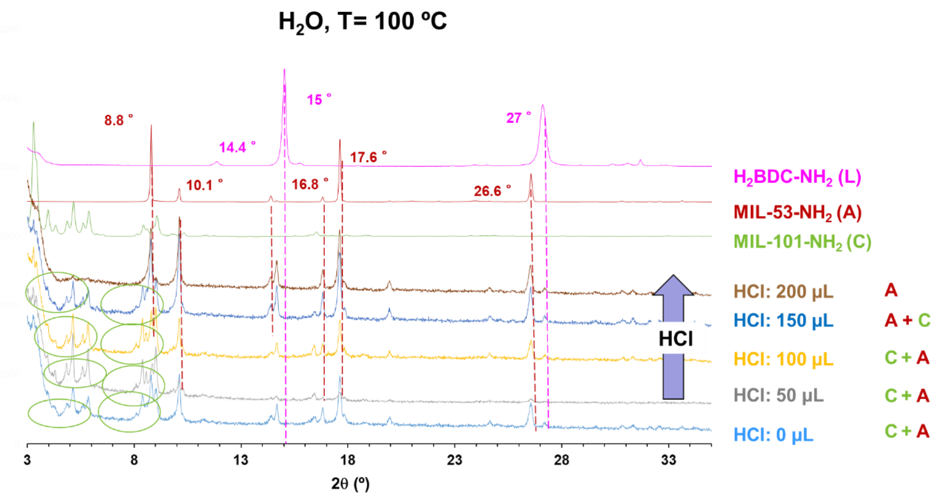

Figure S3. PXRD patterns for the MW investigation of the system $FeCl_3{\cdot}6H_2O/H_2BDC\text{-}NH_2/HCl$ in water (V H2O = 4 mL, T = 100 °C, t = 5 min, [Fe] = 0.05 M) after activation with EtOH, compared to simulated MIL-101-NH_2 (green), MIL-53-NH_2 (red) and H2BDC-NH_2 (pink).

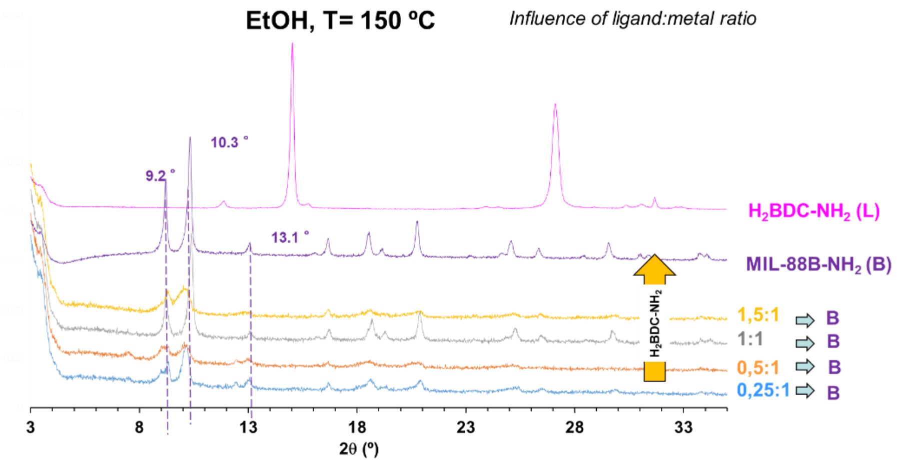

Figure S4. PXRD patterns for the MW investigation of the system $FeCl_3{\cdot}6H_2O/H_2BDC\text{-}NH_2$ in ethanol (V EtOH = 4 mL, T = 150 °C, [Fe] = 0.2 M, t = 5 min) after activation with EtOH, compared to simulated MIL-88B-NH_2 (purple) and $H_2BDC\text{-}NH_2$ (pink).

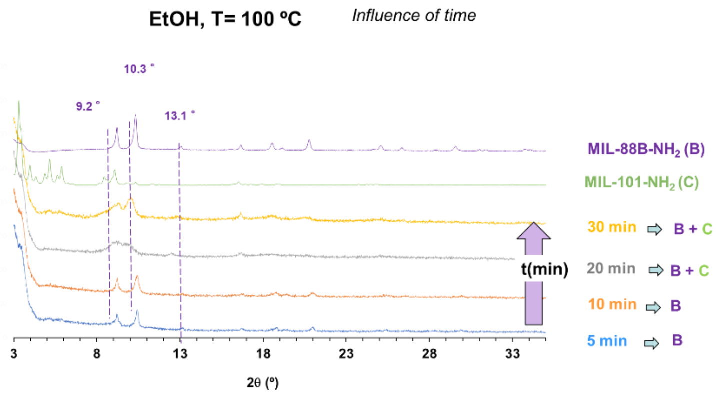

Figure S5. PXRD patterns for the MW investigation of the system $FeCl_3{\cdot}6H_2O/H_2BDC\text{-}NH_2$ in ethanol (V EtOH = 4 mL, T = 100 °C, [Fe] = 0.1 M,) after activation with EtOH, compared to simulated MIL-88B-NH_2 (purple) and MIL-101-NH_2 (green).

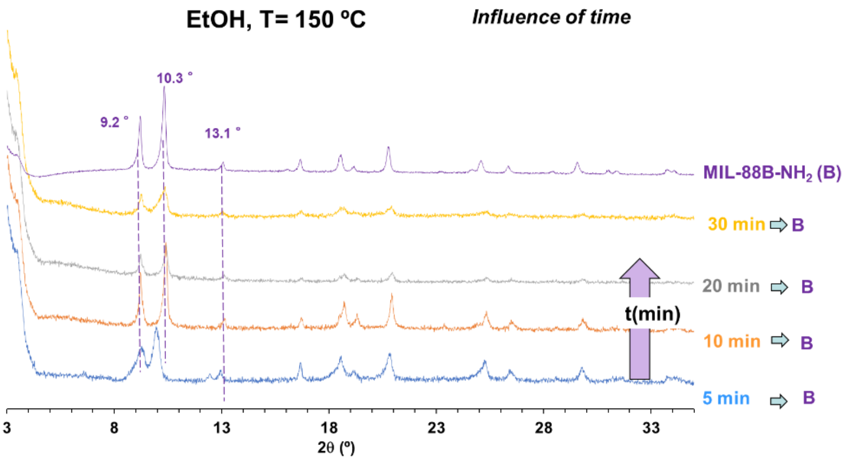

Figure S6. PXRD patterns for the MW investigation of the system $FeCl_3 \cdot 6H_2O/H_2BDC\text{-}NH_2$ in ethanol (V EtOH = 4 mL, T = 150 °C, [Fe] = 0.1 M,) after activation with EtOH, compared to simulated MIL-88B-NH_2 (purple).

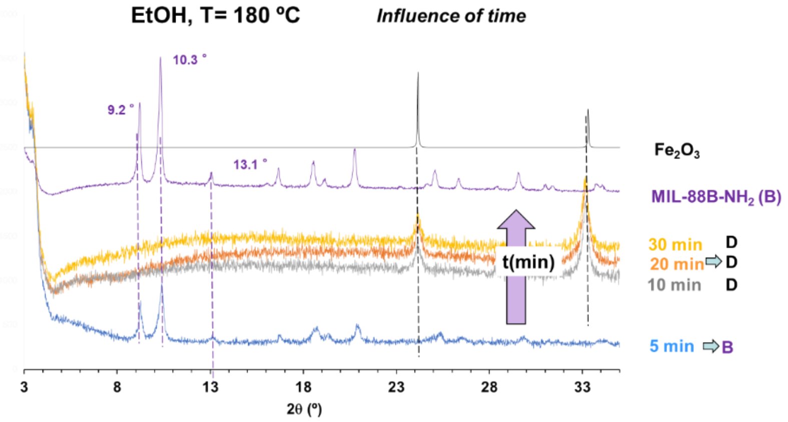

Figure S7. PXRD patterns for the MW investigation of the system $FeCl_3 \cdot 6H_2O/H_2BDC\text{-}NH_2$ in ethanol (V EtOH = 4 mL, T = 180 °C, [Fe] = 0.1 M,) after activation with EtOH, compared to simulated MIL-88B-NH_2 (purple) and Fe_2O_3 (black).

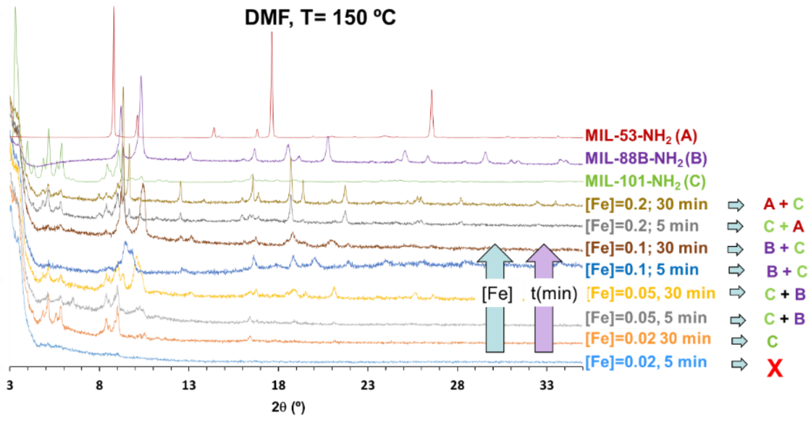

Figure S8. PXRD patterns for the MW investigation of the system $FeCl_3 \cdot 6H_2O/H_2BDC\text{-}NH_2$ in DMF (V DMF = 4 mL, T = 150 °C, ligand:metal = 1:1) after activation with EtOH, compared to simulated MIL-88B-NH_2 (purple) and MIL-101-NH_2 (green).

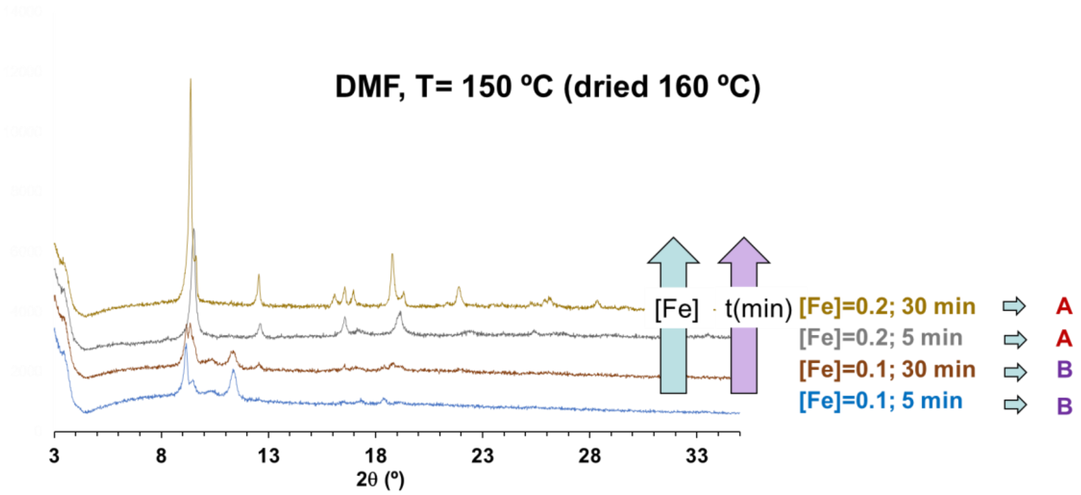

Figure S9. PXRD patterns for the MW investigation of the system $FeCl_3·6H_2O/H_2BDC-NH_2$ in DMF (V DMF = 4 mL, T = 150 °C, ligand:metal = 1:1) after activation with EtOH and dried at 160 °C, for better discriminating between the different Fe-BDC-NH_2 MOF phases.

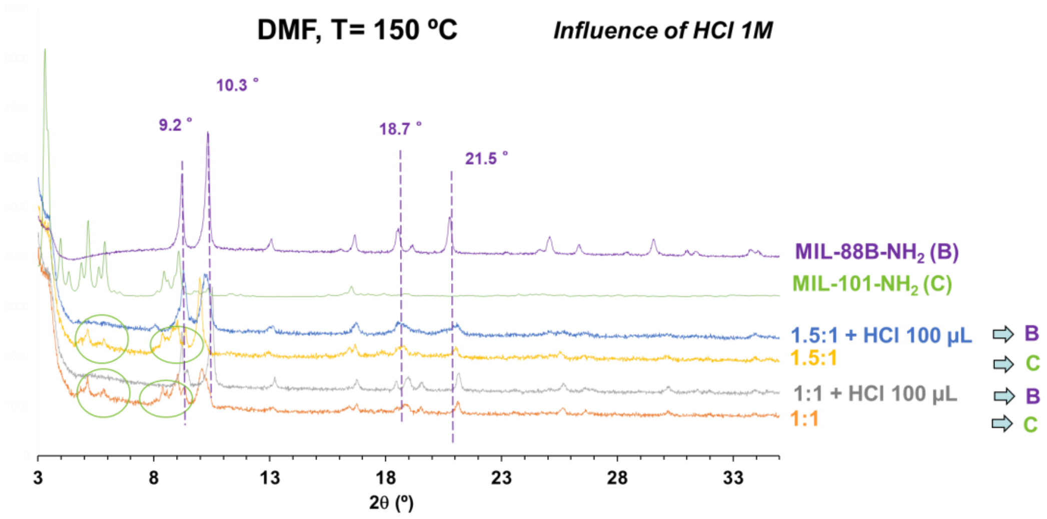

Figure S10. PXRD patterns for the MW investigation of the system $FeCl_3·6H_2O/H_2BDC-NH_2/HCl$ in DMF (V DMF = 4 mL, T = 150 °C, t = 30 min) after activation with EtOH, compared to simulated MIL-88B-NH_2 (purple) and MIL-101-NH_2 (green).

3. Particle size and reaction yield

Table S9. Particle size and reaction yield for the MW investigation of the system $FeCl_3·6H_2O/H_2BDC-NH_2$ in water (V H_2O = 4 mL, T = 100 °C, t = 5 min).

[Fe] (M)	Particle Size (Ø nm)	PdI	Yield (wt.%)	STY ($kg·m^{-3}·d^{-1}$)	Result
0.02	450 ± 200	0.25 ± 0.01	~100(*)	3850	C
0.05	190 ± 70	0.32 ± 0.01	~100(*)	5400	C + A
0.1	450 ± 150	0.20 ± 0.06	~100(*)	8700	C + A
0.2	260 ± 70	0.11 ± 0.02	70	10400	A

* remaining ligand found prior activation due to poor $H_2BDC-NH_2$ solubility in water

Table S10. Particle size and reaction yield for the MW investigation of the system $FeCl_3{\cdot}6H_2O/H_2BDC{-}NH_2$ in water (V H_2O = 4 mL, T= 150 °C, t= 5 min).

[Fe] (M)	Particle Size (Ø nm)	PdI	Yield (wt.%)	STY ($kg{\cdot}m^{-3}{\cdot}d^{-1}$)	Result
0.02	300 ± 85	0.20 ± 0.11	~100(*)	2950	C + A
0.05	300 ± 90	0.37 ± 0.11	~100(*)	3900	A + C
0.1	325 ± 150	0.28 ± 0.03	60	4300	A
0.2	310 ± 120	0.30 ± 0.03	50	7000	A

* remaining ligand found prior activation due to poor $H_2BDC{-}NH_2$ solubility in water

Table S11. Particle size and reaction yield for the MW investigation of the system $FeCl_3{\cdot}6H_2O/H_2BDC{-}NH_2$/HCl in water ([Fe] = 0.05 M, V H_2O = 4 mL, T= 100 °C, t= 5 min).

HCl (μL)	Particle Size (Ø nm)	PdI	Yield (wt.%)	STY ($kg{\cdot}m^{-3}{\cdot}d^{-1}$)	Result
0	240 ± 100	0.35 ± 0.06	~100(*)	8500	C + A
50	480 ± 80	0.41 ± 0.05	~100(*)	8000	C + A
100	500 ± 100	0.50 ± 0.06	~100(*)	7500	C + A
150	590 ± 140	0.41 ± 0.06	~100(*)	7300	A + C
200	320 ± 110	0.39 ± 0.07	~100(*)	6500	A

* remaining ligand found prior activation due to poor $H_2BDC{-}NH_2$ solubility in water

Table S12. Particle size and reaction yield for the MW investigation of the system $FeCl_3{\cdot}6H_2O/H_2BDC{-}NH_2$ in ethanol (V EtOH = 4 mL, T = 150 °C, t = 5 min).

[Fe] (M)	Particle Size (Ø nm)	PdI	Yield (wt.%)	STY ($kg{\cdot}m^{-3}{\cdot}d^{-1}$)	Result
0.02	300 ± 80	0.28 ± 0.12	~100	2050	B
0.05	250 ± 140	0.29 ± 0.09	85	3100	B
0.1	250 ± 80	0.12 ± 0.03	45	3300	B
0.2	210 ± 70	0.10 ± 0.02	40	6200	B

Table S13. Particle size and reaction yield for the MW investigation of the system $FeCl_3{\cdot}6H_2O/H_2BDC{-}NH_2$ in ethanol (V EtOH = 4 mL, T = 150 °C, t= 5 min, [Fe] = 0.2 M).

Ligand:Metal	Particle Size (Ø nm)	PdI	Yield (wt.%)	STY ($kg{\cdot}m^{-3}{\cdot}d^{-1}$)	Result
0.25:1	300 ± 85	0.15 ± 0.07	90	3200	B
0.5:1	290 ± 70	0.07 ± 0.05	60	4700	B
1:1	210 ± 70	0.10 ± 0.02	40	6200	B
1.5:1	270 ± 80	0.12 ± 0.06	35	5600	B

Table S14. Particle size and reaction yield for the MW investigation of the system $FeCl_3 \cdot 6H_2O/H_2BDC\text{-}NH_2$ in ethanol (V EtOH = 4 mL, T = 100 °C, [Fe] = 0.1 M).

Time (min)	Particle Size (Ø nm)	PdI	Yield (wt.%)	STY ($kg \cdot m^{-3} \cdot d^{-1}$)	Result
5	220 ± 80	0.25 ± 0.05	95	16000	B
10	320± 80	0.34 ± 0.08	~100	10700	B
20	240 ± 80	0.28 ± 0.04	~100	6800	C
30	290 ± 70	0.20 ± 0.02	80	3500	C

Table S15. Particle size and reaction yield for the MW investigation of the system $FeCl_3 \cdot 6H_2O/H_2BDC\text{-}NH_2$ in ethanol (V EtOH = 4 mL, T = 150 °C, [Fe] = 0.1 M).

Time (min)	Particle Size (Ø nm)	PdI	Yield (wt.%)	STY ($kg \cdot m^{-3} \cdot d^{-1}$)	Result
5	250 ± 80	0.12 ± 0.03	40	3300	B
10	250 ± 100	0.16 ± 0.08	90	2600	B
20	250 ± 70	0.11 ± 0.01	60	2000	B
30	230 ± 60	0.07 ± 0.04	50	2100	B

Table S16. Particle size and reaction yield for the MW investigation of the system $FeCl_3 \cdot 6H_2O/H_2BDC\text{-}NH_2$ in DMF (V DMF = 4 mL, T = 150 °C, ligand:metal = 1:1).

[Fe] (M)	Time (min)	Particle Size (Ø nm)	PdI	Yield (wt.%)	STY ($kg \cdot m^{-3} \cdot d^{-1}$)	Result
0.02	5	-	-	-	-	X
	30	300 ± 80	0.12 ± 0.09	70	600	C
0.05	5	250 ± 60	0.14 ± 0.09	40	1500	C + B
	30	270 ± 80	0.26 ± 0.09	70	400	C + B
0.1	5	270 ± 70	0.08 ± 0.01	50	3500	B + C
	30	490 ± 150	0.20 ± 0.02	70	2300	B + C
0.2	5	230 ± 80	0.2 ± 0.01	20	6000	C + A
	30			45	3800	A + C

Table S17. Particle size and reaction yield for the MW investigation of the system $FeCl_3 \cdot 6H_2O/H_2BDC\text{-}NH_2/HCl$ in DMF (V DMF = 4 mL, T = 150 °C, t = 30 min, [Fe] = 0.05).

Ligand:Metal	HCl (µL)	Particle Size (Ø nm)	PdI	Yield (wt.%)	STY ($kg \cdot m^{-3} \cdot d^{-1}$)	Result
1:1	0	270 ± 80	0.26 ± 0.09	70	400	C + B
	100	293 ± 100	0.34 ± 0.06	90	500	B
1.5:1	0	500 ± 150	0.18 ± 0.02	70	450	C + B
	100	600 ± 130	0.20 ± 0.09	~100	600	B

4. TEM

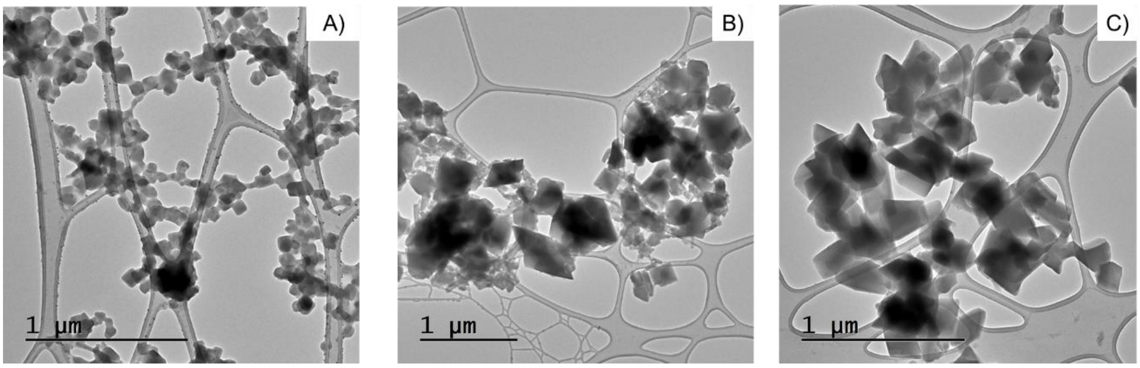

Figure S11. TEM micrographs for PXRD for the MW investigation of the system $FeCl_3{\cdot}6H_2O/H_2BDC\text{-}NH_2/HCl$ in water (V H_2O = 4 mL, t = 5 min) after activation with EtOH: (**A**) T = 100 °C, HCl 0.1 M = 0 μL; (**B**) T = 100 °C, HCl 0.1 M = 100 μL; (**C**) T = 150 °C, HCl 0.1 M = 0 μL.

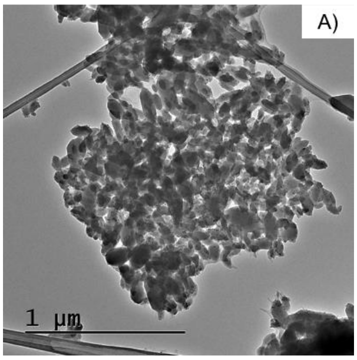

Figure S12. TEM micrographs for PXRD for the MW investigation of the system $FeCl_3{\cdot}6H_2O/H_2BDC\text{-}NH_2$ in ethanol (V EtOH = 4 mL, t = 5 min T = 100 °C, [Fe] = 0.1 M) after activation with EtOH scale bar = 1 μm.

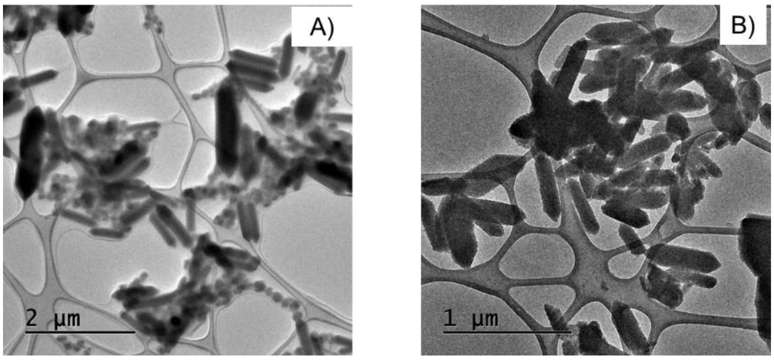

Figure S13. TEM micrographs for PXRD for the MW investigation of the system $FeCl_3{\cdot}6H_2O/H_2BDC\text{-}NH_2/HCl$ in DMF (V DMF = 4 mL, t = 30 min, T = 100 °C, ligand:metal = 1:1) after activation with EtOH: (**A**) HCl 0.1 M = 0 μL (scale bar = 2 μm); (**B**) HCl 0.1 M = 100 μL (scale bar = 1 μm).
